# Global DNA Methylation Profiling Reveals Differentially Methylated CpGs between Salivary Gland Pleomorphic Adenomas with Distinct Clinical Course

**DOI:** 10.3390/ijms23115962

**Published:** 2022-05-25

**Authors:** Katarzyna Kiwerska, Ewelina Kowal-Wisniewska, Adam Ustaszewski, Ewelina Bartkowiak, Malgorzata Jarmuz-Szymczak, Malgorzata Wierzbicka, Maciej Giefing

**Affiliations:** 1 Institute of Human Genetics, Polish Academy of Sciences, Strzeszynska 32, 60-479 Poznan, Poland; ewelina.kowal@igcz.poznan.pl (E.K.-W.); adam.ustaszewski@igcz.poznan.pl (A.U.); malgorzata.jarmuz-szymczak@igcz.poznan.pl (M.J.-S.); otosk2@gmail.com (M.W.); maciej.giefing@igcz.poznan.pl (M.G.); 2 Department of Tumor Pathology, Greater Poland Cancer Centre, Garbary 15, 61-866 Poznan, Poland; 3 Department of Hematology and Bone Marrow Transplantation, Poznan University of Medical Sciences, Szamarzewskiego 84, 60-569 Poznan, Poland; 4 Department of Otolaryngology and Laryngological Oncology, Poznan University of Medical Sciences, Przybyszewskiego 49, 60-355 Poznan, Poland; ewelina.anna.bartkowiak@gmail.com

**Keywords:** salivary gland pleomorphic adenoma, fast-growing tumors, slow-growing tumors, DNA methylation, CpG, bisulfite pyrosequencing

## Abstract

Pleomorphic adenomas (PAs) are the most frequently diagnosed benign salivary gland tumors. Although the majority of PAs are characterized by slow growth, some develop very fast and are more prone to recur. The reason for such differences remains unidentified. In this study, we performed global DNA methylation profiling using the Infinium Human Methylation EPIC 850k BeadChip Array (Illumina) to search for epigenetic biomarkers that could distinguish both groups of tumors. The analysis was performed in four fast-growing tumors (FGTs) and four slow-growing tumors (SGTs). In all, 85 CpG dinucleotides differentiating both groups were identified. Six CpG tags (cg06748470, cg18413218, cg10121788, cg08249296, cg18455472, and cg19930657) were selected for bisulfite pyrosequencing in the extended group of samples. We confirmed differences in DNA methylation between both groups of samples. To evaluate the potential diagnostic accuracy of the selected markers, ROC curves were constructed. We indicated that CpGs included in two assays showed an area under the curve with an acceptable prognostic value (AUC > 0.7). However, logistic regression analysis allowed us to indicate a more optimal model consisting of five CpGs ((1) cg06748470, (2) cg00600454, (3) CpG located in chr14: 77,371,501–77,371,502 (not annotated in GRCh37/hg19), (4) CpG2 located in chr16: 77,469,589–77,469,590 (not annotated GRCh37/hg19), and (5) cg19930657) with AUC > 0.8. This set of epigenetic biomarkers may be considered as differentiating factors between FGT and SGT during salivary gland tumor diagnosis. However, this data should be confirmed in a larger cohort of samples.

## 1. Introduction

Salivary gland neoplasms constitute a very heterogeneous group of tumors, both in terms of histology and anatomical location. According to the 2017 WHO classification of head and neck tumors, over 40 different malignant and benign types of salivary gland tumors can be distinguished [1]. These tumors may arise in major salivary glands, i.e., in the parotid, submandibular, and sublingual glands, but they also affect minor salivary glands. Most of these tumors are benign and their predominant location is the parotid gland [2,3]. Among them, pleomorphic adenomas (PAs) appear as the most frequent, with an increasing rate in recent years [4,5,6]. The surgical resection sparing the facial nerve remains a standard treatment option for PA. Depending on the site and the extent of the tumor, the surgical techniques include extracapsular resection, partial superficial parotidectomy, superficial parotidectomy, subtotal parotidectomy, or total parotidectomy [7,8]. In cases of submandibular tumor gland, dissection is performed.

Pleomorphic adenomas are composed of epithelial and myoepithelial cells differentially arranged in a variable background stroma, depending on the case [1,4]. PAs are usually slow-growing tumors, with a firm, mobile, well-circumscribed mass. Their growth is rather painless and asymptomatic; therefore, they may remain unrecognized for a long period of time [9]. Their malignant transformation is a very rare event [10,11]. Nevertheless, there is a subgroup of PAs that break out of these characteristics, showing significantly diverse clinical behavior. Such tumors grow faster, and patients may observe the enlarging thickening in the neck/lower jaw area even within a few months. The observed differences were recently described by Piwowarczyk et al., who distinguished three groups of PAs: (I) fast-growing, (II) normal/stable, and (III) slow-growing based on the clinical and radiological data of 636 PAs patients [12]. The reason for such diversity in the clinical behavior is so far unknown. At the stage of PA diagnosis, neither the pathologist (based on fine needle aspiration biopsy (FNA)), nor the clinician (based on patients’ history, clinical examination, and imaging) is able to categorize the tumor, i.e., to predict its fast- or slow-growing nature. Although the level of malignant transformation rate of PAs is not high, they may recur, predominantly within the fast-growing group, which results in the necessity of additional surgery and inconvenience for the patient [12]. The relapse occurrence depends mainly on the surgeon’s skills; however, adverse PA findings, such as high tumor growth rate, multifocal character, and lack of encapsulation, and its ability for malignant transformation depend on the tumor biology.

The most comprehensive information concerning PAs is derived from histological studies [13]; however, these data are not sufficient to predict the rate of tumor growth. On the other hand, little is known about the genetic background of PA’s development. Although it was shown that *PLAG1* and *HDMD2* rearrangements are a characteristic feature of these tumors, these findings have not been associated with a differential course of PAs [4]. Therefore, the recognition of genetic characteristics of both groups would be helpful in the decision-making process concerning PA patients.

The lack of recurrent genetic events in PAs triggered us to analyze for epigenetic alterations in these tumors. DNA methylation is known to be involved in tumor development [14]. The significant role of this process during carcinogenesis was previously indicated for promoter regions of various tumor-suppressor genes, which are frequently affected by hypermethylation, which results in the loss of their protective, anticancer functions [14,15]. In recent years, several DNA methylation biomarkers have been introduced in the early diagnostic process of different cancers [16]. Moreover, the utility of DNA methylation was also indicated in the development of various nonmalignant diseases [17]. 

In the present study, we harnessed DNA methylation analysis in the process of discriminating between fast-growing and slow-growing salivary gland PAs. We performed global DNA methylation profiling of these tumors in order to establish methylation biomarkers that could distinguish both groups. We assume that such a set of markers could be potentially applied in the early diagnostics of PA patients during, e.g., fine needle aspiration biopsy of the gland. It could also serve as a guide for a surgeon to choose a more extensive procedure (parotidectomy) rather than a sparing one (extracapsular dissection). Such an approach allowed recently for the identification of several methylation biomarkers in other tumors [18,19]. 

To our knowledge, this is the first whole-genome study concerning DNA methylation biomarkers in PAs.

## 2. Results

**Figure 1:**
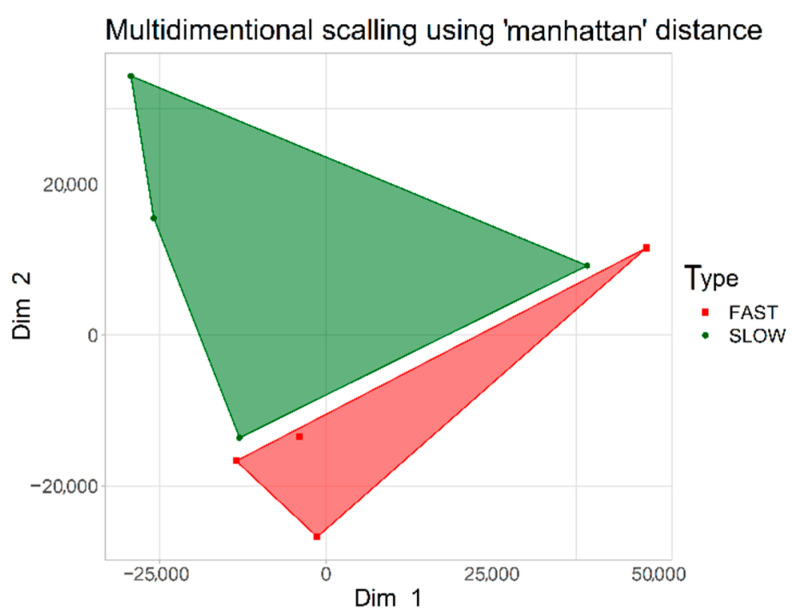
Visualization of MDS analysis using the “Manhattan” method of distance calculation. Two analyzed cohorts are shown: fast-growing salivary gland tumors (red) and slow-growing salivary gland tumors (green).

The MDS analysis revealed clear separation of the studied groups (FGT vs. SGT; Figure 1), serving as a proof of principle that DNA methylation can be used to distinguish these two groups of tumors. However, the relatively close distance between the groups is indicative of minor differences in their methylome.

### 2.1. CpG Tags Selection Based on DNA Methylation Profiling

**Figure 2:**
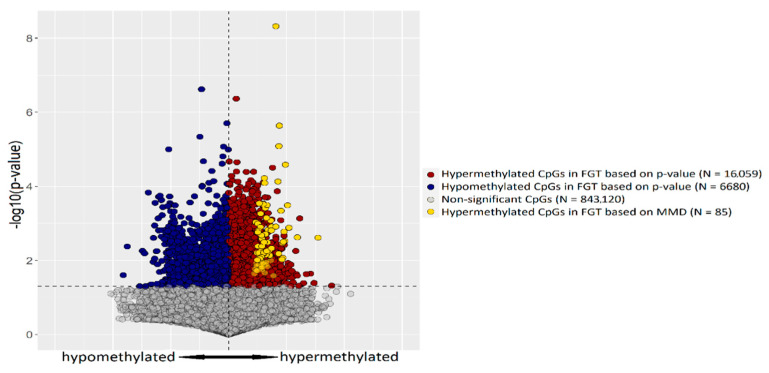
Volcano plot of DNA methylation data derived from microarray analysis of FGT (*n* = 4) and SGT (*n* = 4) samples. Red and blue dots represent hyper- and hypomethylated CpG sites in FGTs, respectively, gray dots represent nonsignificant CpG sites. In all, 85 CpGs hypermethylated in FGT, selected based on MMD, are shown as yellow dots in the background of all significantly hypermethylated tags.

To detect differentially methylated regions (DMRs) distinguishing FGTs from SGTs, we selected CpG tags located on autosomal chromosomes, for which the difference in DNA methylation between fast- and slow-growing tumors exceeded 0.4 (i.e., 40%). With this approach, we obtained 215 CpG dinucleotides. This list was further shortened to 85 CpG sites, when the SD criterion was included. The list of these CpG tags, together with their chromosome position, mean methylation value, mean methylation difference between the analyzed groups, and standard deviation is presented in Supplementary Table S1. The distribution of the selected 85 CpGs in relation to all tags indicates that the selected tags are included in the group of CpGs significantly hypermethylated in the fast-growing tumors and is, therefore, a proof of concept of the applied approach (Figure 2).

**Figure 3:**
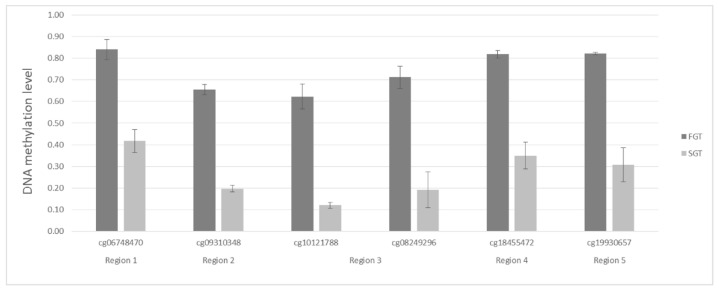
Mean methylation levels between fast-growing (dark gray) and slow-growing (light gray) tumors for the six CG tags selected based on the results of the Illumina Infinium Human Methylation EPIC BeadChip Array.

Afterward, each of the 85 tags was checked using the array data, for the presence of adjacent tags in its close proximity (~500 bp). Only regions, where additional tag(s) showed a trend toward higher methylation in FGT compared to SGT were analyzed. With this approach, we selected six CpG tags, cg06748470, cg18413218, cg10121788, cg08249296, cg18455472, and cg19930657, to be the most promising biomarkers differentiating both groups (Supplementary Table S2). Mean methylation differences for the selected CpGs are presented in Figure 3. Two of the tags, cg10121788 and cg08249296, are located close to each other (at 128 bp distance) in the regulatory region of *ADAMTS18* gene. Therefore, in the end, five chromosomal regions were selected for further testing by bisulfite pyrosequencing.

### 2.2. Bisulfite Pyrosequencing Confirms Differential DNA Methylation between FGT and SGT Samples

The assays were designed with PyroMark Assay Design Software 2.0 (Qiagen, Hilden, Germany) to assess the methylation level in individual CpG sites. We designed five assays to amplify the sequences containing primarily CpG tags selected from the methylation array (i.e., cg06748470, cg18413218, cg10121788, cg18455472, and cg19930657). However, due to the presence of rs62043630 SNP in cytosine of cg08249296, we decided to exclude this sequence tag from further analysis. Moreover, as a result of inter-sequence homopolymer presence in assay 2, we were not able to analyze the results obtained for the cg18413218 tag, providing only results obtained for its adjacent CpG tags. The chromosomal location of all analyzed CpGs in each pyrosequencing assay is given in Supplementary Table S3. 

**Figure 4:**
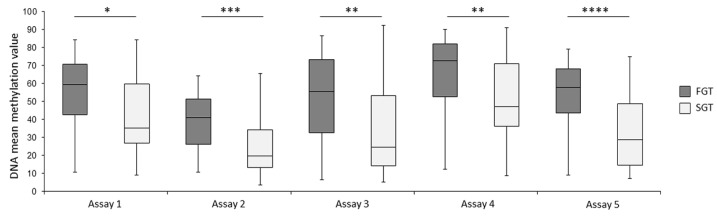
Mean methylation differences between fast-growing (dark gray) and slow-growing (light gray) tumors for each assay performed by DNA bisulfite pyrosequencing. * *p* < 0.05, ** *p* < 0.001, *** *p* < 0.0001, **** *p* < 0.00001.

We found that the mean methylation level for each of the analyzed CpG sites was significantly higher in FGT samples in comparison to that for the SGT samples (Table 1). The mean methylation values for the consecutive assays were as follows: assay 1—53.8% FGT vs. 42.2% SGT; assay 2—38.7% FGT vs. 25.2% SGT; assay 3—51.5% FGT vs. 33.7% SGT; assay 4—66.5% FGT vs. 51.8% SGT; assay 5—52.4% FGT vs. 32.1% SGT samples. Overall, the mean methylation difference between FGT and SGT in the given assays ranged from 11.6% to 20.3%. With the use of nonparametric Mann–Whitney test for unpaired data, we showed that not only each individual CpG tag but also each assay differentiated both groups of PA patients with significant differences (Table 1, Supplementary Figure S1, Figure 4).

### 2.3. Individual CpG Dinucleotides as Potential Biomarkers

Since pyrosequencing confirmed the results provided by the methylation array, we attempted to check the potential diagnostic utility of the individual CpGs as well as of the performed assays. For this purpose, ROC curves for each analyzed CpG tag were generated to establish a relationship between the sensitivity and specificity of this potential diagnostic approach (Supplementary Figure S2). The sensitivity, specificity, area under the curve, statistical significance of DeLong’s test, methylation cutoff, positive predictive value, and negative predictive value are shown in Table 2.

The sensitivity of particular CpG dinucleotides ranged from 0.69 (CpG2 in assay 3 and CpG2 in assay 4) to 0.81 (CpG2 in assay 2 and CpG1 in assay 4), and specificity was between 0.59 (CpG1 in assay 4) and 0.72 (CpG2 in assay 3 and CpG1 in assay 5). The smallest area under the curve was 0.63 (CpG4 in assay 1), and the highest was 0.76 (CpG1 in assay 5). Using DeLong’s test, we proved that, for each individual CpG, the AUC differed significantly from the fully random classifier (AUC = 0.5). 

The sensitivity of pyrosequencing assays ranged between 0.75 and 0.83, and specificity ranged between 0.56 and 0.72. For assays number 2 and 5, the AUC values were above 0.7. For assays 1, 3, and 4 the AUC values were lower, between 0.6 and 0.7. The probability of proper qualification of patients to the fast-growing group (PPV) was between 0.64 and 0.81, and for the slow-growing group (NPV), it was between 0.68 and 0.79 (Table 2). 

**Figure 5:**
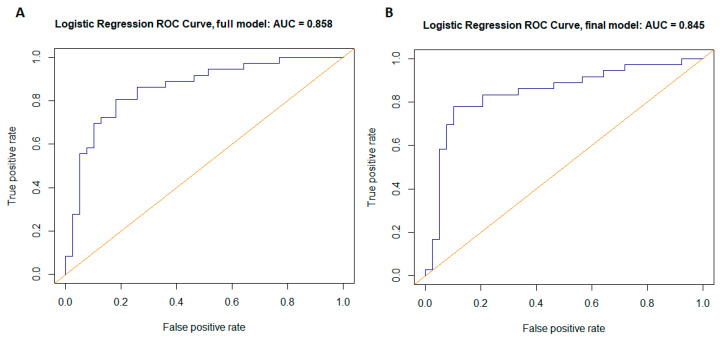
Visualization of ROC curves representing two logistic regression models: (**A**) full model (all explanatory variables, i.e., all CpG dinucleotides included) and (**B**) final model obtained by stepwise approach (AIC) including only selected CpG dinucleotides.

Due to the low AUC, specificity, and sensitivity of models with individual, particular CpG dinucleotides as predictor variables, two logistic regression models were prepared: (1) full model with all explanatory variables included and (2) final model obtained by stepwise approach based on AIC (Table 3 and Figure 5).

The final model consists of the five following biomarker CpG dinucleotides as a result of the stepwise selection: CpG1 (cg06748470) and CpG5 (cg00600454) of assay 1, CpG2 (chr14: 77,371,501–77,371,502 (not annotated in GRCh37/hg19 database)) of assay 2, CpG2 (chr16: 77,469,589–77,469,590 (not annotated in GRCh37/hg19 database)) of assay 3, and CpG1 (cg19930657) of assay 5. The main advantage of this model is the reduced number of explanatory variables: 5 CpG dinucleotides instead of 13 CpGs in the full model. Pyrosequencing of lower number of CpGs may simplify a potential test and speed up early differential diagnosis of the patients. Moreover, the bootstrap approach in the final model revealed lower potential bias than in the full model with all 13 variables (Table 3). After bias correction, the AUC of the final model was >80% in comparison to that of the full model, <75%. It shows the final model as the better classifier regarding distinguishing between slow- and fast-growing tumors.

## 3. Discussion

Recent decades have brought a marked increase in the incidence of salivary gland tumors [5,12,20]. Among them, pleomorphic adenomas are the most frequent, and as previously observed, their development may vary in the context of tumor growth rate and malignant transformation [12,21]. On one hand, at the stage of initial diagnosis, both pathologists as well as clinicians are not able to assign the tumor to the fast-growing or slow-growing cohort. On the other hand, some PAs should be timely removed, other scheduled for removal after a longer time period, or some exceptionally even recommended for the “wait and see” policy. Moreover, the accurate risk stratification also cannot be predicted based on histopathological assessment of the specimen. There are currently no recommendations to perform any additional staining that could help to classify PA as either SGT or FGT. Although PA shows a wide spectrum of histopathological features with variation in both epithelial and stromal components, the final diagnosis does not reflect this diversity and is reduced to salivary gland pleomorphic adenoma.

Given that the etiology of this disease as well as its genetic background is still widely unrecognized, we took a look at the epigenetic landscape of pleomorphic adenomas with different clinical courses. We performed genome-wide methylation analysis and bisulfite pyrosequencing validation at selected chromosomal regions (specific CpG) in order to identify biomarkers potentially discriminating between fast- and slow-growing PAs. Such an approach is novel in the context of PA as the previous reports have rather examined selected genes as potentially methylated in PA and/or the subsequent CaexPA and were not related to the differential course of PA [22,23,24]. In contrast, in our recent paper, we revealed differences in p16Ink4a protein expression between FGT and SGT PAs and demonstrated that its overexpression is connected to PA proliferation and subsequent malignant transformation to CaexPA [21].

Both groups analyzed in our study had the same pathomorphological diagnosis, i.e., pleomorphic adenoma; however we demonstrate that it is possible to discriminate between FGT and SGT samples based on DNA methylation profiles (Figure 1). Regardless of the fact that the observed methylome differences were minor and as such were markedly smaller than those usually found when comparing cancer samples against normal samples, this observation constitutes the proof of principle of the approach proposed in this study.

Based on global DNA methylation analysis, we indicated six selected CpG dinucleotides to be the most promising biomarkers differentiating both groups (Supplementary Table S2), which was further confirmed by DNA bisulfite pyrosequencing performed in the enlarged groups of samples. Due to the specificity of the collected material (lack of the corresponding normal samples), we focused only on finding epigenetic biomarkers differentiating both analyzed groups instead of searching for characteristic genes that are hypermethylated during the faster course of PA development. A similar approach for identifying biomarkers has been already applied in tumor studies [25].

Regardless of tissue similarities between the analyzed groups in our study, we were able to indicate significantly higher methylation levels in FGTs in comparison to SGTs for both consecutive assays as well as for particular CpG tags (Table 1). Encouraged by this result, we evaluated the potential diagnostic utility of the assays. Only two out of five assays (No. 2 and No. 5) showed an acceptable prognostic value (AUC > 0.7) [26]. Therefore, to assess whether the combination of all 13 CpG dinucleotides (full model with all explanatory variables included in all five assays) will allow to create more accurate prognostic model, logistic regression analysis was performed. With this model, higher AUC, sensitivity, and specificity in comparison to the individual CpG sites were shown (Table 3; Figure 5A). However, the subsequent bootstrap approach revealed relatively high bias regarding this full model, negatively influencing the final AUC value. The AIC approach, limited to the optimal explanatory variables with the highest influence on the response variable (fast or slow character of the tumor) revealed the optimal model with similar accuracy to the original variant but with lower bias. In addition, the higher specificity (0.9) should be noted here. Hence, the obtained final model may be considered as a potential epigenetic biomarker model that may be used to diagnose a particular salivary gland tumor as fast growing or slow growing. The proposed panel is based on the methylation of five CpG dinucleotides: (1) cg06748470, (2) cg00600454, (3) CpG located in chr14: 77,371,501–77,371,502 (not annotated in GRCh37/hg19 database), (4) CpG2 located in chr16: 77,469,589–77,469,590 (not annotated GRCh37/hg19 database), and (5) cg19930657 (Table 3; Figure 5B). A similar approach was recently applied for miRNA biomarkers in head and neck squamous cell carcinomas [27]. 

Nevertheless, our research has several limitations. The model was built with the use of a small number of data (75 samples); therefore, it should be verified in the larger cohort of pleomorphic adenoma patients. Additionally, to observe whether the classification of SGT and FGT makes a relevant difference with regard to follow up, PA patients should be observed for at least 10 years before the final conclusion can be drawn. In the studied group, for some patients, the follow-up observation did not exceed two years, but was still ongoing. None of the patients developed recurrence or CaexPA so far.

To conclude, the study has the potential to be efficiently translated into clinical practice. The proposed panel based on DNA methylation can be applied on the oligobiopsy specimens prior to surgery in the decision-making process. Our results indicate that the molecular assessment may be invaluable after a relevant period of observation; however, for now, it should rather be considered as a helpful supplement in the diagnostic process. In addition, it seems that searching for other genetic alterations differentiating FGTs and SGTs is highly justified.

## 4. Materials and Methods

### 4.1. Study Group

The study group consisted of 75 patients diagnosed and treated for PA in the Department of Otolaryngology and Laryngological Surgery, Poznan University of Medical Sciences, between 2015 and 2020. The study was approved by the Ethical Board of the Poznan University of Medical Sciences (approval 721/18). Patients were enrolled to the study when they initially presented in the outpatient department and qualified for surgery. At that time, based on patients’ history, clinical examination, and imaging, they were divided into SGT or FGT, and written consents for molecular examination were collected. Each patient was classified with either a fast-growing tumor (FGT) or a slow-growing tumor (SGT) tumor based on the anamnesis, including symptoms onset and ultrasound examination of the tumor by two independent ENT specialists (E.B. and M.W.). The inclusion criteria were based on a recent publication with slight modification resulting from the lack of patients fulfilling the temporal criteria of slow-growing tumors (appearance > 10 years) [12]. The aim was to distinguish fast-growing PAs from the remaining ones, growing either slow or normal. Therefore, herein, slow- and normal-growing tumors were considered together as one group, namely SGT. Ultimately, FGTs (*n* = 36) were characterized by anamnesis <3 years; >5% growth of the tumor size within six months; multi-polycyclic outline, heterogeneous echostructure, and loss of capsule echogenicity in the radiological investigation. For SGTs (*n* = 39), the inclusion criteria were as follows: anamnesis > 3 years, <5% growth of the tumor size within six months, well-visualized tumor capsule in radiological investigation, and tumor homogeneity. 

From each patient, a small fragment of tumor tissue was taken during the surgery and kept frozen at −80 °C until DNA isolation. The remaining tissue was passed for routine histopathological examination, where pathologists confirmed the diagnosis of PA. Thereafter, the patient was ultimately classified to the study. DNA isolation was conducted using standard phenol/chloroform procedure. The data concerning age, sex, and applied treatment of PA patients are presented in Supplementary Table S4. 

### 4.2. Global DNA Methylation Profiling

From the group of 75 PA samples, 4 FGTs and 4 SGTs were selected for global DNA methylation profiling, performed with the use of a high-resolution Infinium Human Methylation EPIC 850k BeadChip Array (Illumina Inc. San Diego, CA, USA) in the Atlas Biolabs Company (Berlin, Germany). This array provides information on the DNA methylation status of 865,859 CpG dinucleotides (called CpG sequence tags) dispersed throughout the whole human genome. The obtained data were subsequently preprocessed using the minfi package with functional normalization (funnorm) followed by noob background correction [28,29,30]. As a result, β-values, i.e., methylation levels of each sample at each CpG dinucleotide, ranging from 0 (fully unmethylated) to 1 (fully methylated) were obtained. β-values were derived from the following calculation: ratio of the probe’s methylated signal intensity to the sum of the methylated and unmethylated probe signal intensities [31].

### 4.3. Selection of CpG Tags Differentiating Fast-Growing from Slow-Growing PAs

The assumption of the methylome analysis was to indicate CpG sites with a significantly higher DNA methylation level in the FGTs in comparison to the SGTs. Mean methylation values (MMVs) for fast-growing PAs (*n* = 4) and for slow-growing PAs (*n* = 4) were calculated for each of the 865,859 CpG tags. To avoid any gender-specific methylation bias, CpGs located on chromosomes X and Y were excluded. Afterward, the mean methylation difference (MMD) between the FGT and the SGT was established for each sequence tag. Only CpG dinucleotides for which the difference between the FGT mean β value and the SGT mean β value exceeded 0.4 were further considered. Moreover, in both analyzed groups, the standard deviation (SD) was determined, and CpG dinucleotides with SD exceeding 0.2 (representing diversity in DNA methylation above 20% between samples in the compared groups) in either of the groups were excluded.

### 4.4. Validation of DNA Methylation by Bisulfite Pyrosequencing

To perform DNA methylation analysis of the selected regions in the extended group of patients (36 FGTs vs. 39 SGTs), bisulfite pyrosequencing was performed. At first, assays for each selected region were designed with the use of PyroMark Assay Design Software 2.0 (Qiagen, Hilden, Germany). Each pyrosequencing assay included two primers for amplicon preparation (one of the primers was biotin-labeled at the 5′ end) and one sequencing primer. All primers were purchased from the Genomed Company (Warsaw, Poland; Supplementary Table S5).

Briefly, 500 ng of DNA was bisulfite treated using an EZ DNA Methylation—Gold^TM^ Kit (Zymo Research, Irvine, CA, USA), according to the manufacturer’s protocol. PCR was performed with the use of a PyroMark PCR kit (Qiagen, Hilden, Germany). The PCR reaction mixture’s composition and the reaction conditions are presented in Supplementary Table S6. The specificity of the PCR reaction was checked by electrophoresis in 1.8% SimplySafe stained agarose gel (EurX, Gdansk, Poland) and visualized under UV light. All assays included conversion control and were designed using the PyroMarkQ48 Autoprep 2.4.2 Software (Qiagen, Hilden, Germany). Pyrosequencing was performed with the use of PyroMark Q48 Advanced CpG Reagents (Qiagen, Hilden, Germany) and a PyroMark Q48 Autoprep sequencer (Qiagen, Hilden, Germany) according to standard manufacturer’s protocol. In addition to the tested samples, each analysis included fully methylated and unmethylated controls (Sigma-Aldrich, Saint Louis, MO, USA). As a result, the mean methylation level of the analyzed CpGs within the region of interest (ranged 0–100%) was obtained in each analyzed sample. Overall, in individual assays, DNA methylation was measured in one to five CpG sites.

### 4.5. Statistical Analysis

To visualize the global comparison between studied samples (4 FGTs and 4 SGTs), multidimensional scaling (MDS) with Manhattan distance was used. A volcano plot was created with the ggplot2 R package using normalized microarray methylation data [32]. The differences in the methylation levels for particular CpGs between the FGT and SGT groups were determined with the use of a nonparametric Mann–Whitney Test for unpaired data [33]. Since our goal in this study was to determine hypermethylated regions exclusive in FGT compared to SGT, the one-sided test was used. The statistical significance threshold was set at *p* < 0.05. Likewise, the difference in methylation level was also assessed for each pyrosequencing assay by calculating DNA mean methylation values of all CpG dinucleotides included in the given assay between analyzed groups (36 FGTs and 39 SGTs).

To evaluate the potential diagnostic accuracy of selected methylation markers, receiver operating characteristic (ROC) curves were constructed separately for each CpG dinucleotide and for the given assay. The ROC curves were obtained by plotting the true positive rate (sensitivity) on the y-axis as a function of false positive rate (1-specificity) on the x-axis [26]. By calculating the area under the curve (AUC), we measured the power of particular CpGs as well as of whole assays in discriminating between the analyzed group of samples. While an AUC of 1.0 reflects a perfectly accurate test, values above 0.7 are considered acceptable [26]. Moreover, by using the DeLong test we verified whether the AUCs of the analyzed CpGs and assays differed significantly from the fully random classifier (AUC = 0.5). 

Afterward, the stepwise logistic regression was used in order to determine the subset of selected CpG dinucleotides as optimal predictive model. To obtain such a model, Akaike’s Information Criteria (AIC) were used. This method allows the determination of the best model (with the highest prediction accuracy) with the fewest number of independent variables, i.e., CpG dinucleotides, which is crucial for possible diagnostic use in the future. Due to the small number of observations, this selection was performed using the model built on a full dataset, i.e., 75 observations and 13 explanatory variables/predictors (full model, i.e., 13 CpG dinucleotides analyzed together using five pyrosequencing assays). The final model with the lowest AIC coefficient was built using selected predictors and was evaluated against its accuracy and prediction power. The evaluation consisted of ROC analysis and 1000 simulations of predictions using training and test data split in an 8:2 ratio. Finally, to add bias correction to the AUC of the discussed models, the bootstrap approach was applied. The analysis and calculations were performed using the following R packages: stats, pROC, and caret [34,35,36,37].

The highest sensitivity and specificity values obtained from selected classifiers indicated the optimal cutoff points regarding each selected methylation marker. These cutoff points denote a particular methylation level of the selected marker as a potential diagnostic threshold. By this approach, based on the methylation level of the analyzed tumor, a sample may be classified as a fast-growing subtype. Eventually, positive predictive value (PPV) and negative predictive value (NPV) were calculated in order to establish the probability that the patients were adequately classified to the groups both for those who truly have a fast-growing tumor (PPV) and for those who truly do not have a fast-growing tumor (NPV). 

## Figures and Tables

**Table 1:** The results of DNA bisulfite pyrosequencing analysis.

Pyrosequencing Assay No.	CpG No.	CpG Name ^a^	MMV ^c^ (FGT)	MMV ^c^ (SGT)	MMD ^d^ (MMF—MMS)	*p* Value
**1**	CpG1	cg06748470	50.8	38.9	12.0	0.012451
	CpG2	N/A ^b^	67.0	54.2	12.9	0.017414
	CpG3	cg09774749	48.9	38.0	10.9	0.015255
	CpG4	cg15208832	59.9	47.9	12.0	0.025001
	CpG5	cg00600454	42.1	32.1	10.0	0.022008
	Mean CpG1-CpG5		53.8	42.2	11.6	0.017372
**2**	CpG1	cg09310348	41.6	26.2	15.4	0.000422
	CpG2	N/A ^b^	35.8	24.2	11.6	0.000216
	Mean CpG1-CpG2		38.7	25.2	13.5	0.000267
**3**	CpG1	N/A ^b^	48.6	31.8	16.8	0.002461
	CpG2	N/A ^b^	56.2	38.8	17.3	0.003937
	CpG3	cg10121788	49.8	30.5	19.3	0.002914
	Mean CpG1-CpG3		51.5	33.7	17.8	0.003122
**4**	CpG1	cg14066163	73.5	59.3	14.2	0.002378
	CpG2	cg18455472	59.5	44.3	15.2	0.002297
	Mean CpG1-CpG2		66.5	51.8	14.7	0.002068
**5 ^e^**	CpG1	cg19930657	52.4	32.1	20.3	0.000048

^a^ Human GRCh37/hg19 assembly; ^b^ CpG dinucleotide not annotated in GRCh37/hg19 database; ^c^ MMV—mean methylation value; ^d^ MMD—mean methylation difference; ^e^ adjacent CpG tags with a similar methylation pattern are located outside the sequencing range of the pyrosequencing assay.

**Table 2:** Individual CpG and assay performance based on ROC curves.

Pyrosequencing Assay No.	CpG No.	CpG Name ^a^	Sensitivity	Specificity	AUC ^c^	*p* Value (DeLong’s Test)	Cutoff ^d^	NPV ^e^	PPV ^f^
**1**	CpG1	cg06748470	0.75	0.69	0.65	0.02762	41.95	0.75	0.69
	CpG2	N/A ^b^	0.78	0.67	0.64	0.03868	56.81	0.76	0.68
	CpG3	cg09774749	0.75	0.67	0.65	0.03353	38.41	0.74	0.68
	CpG4	cg15208832	0.72	0.69	0.63	0.05576	51.76	0.73	0.68
	CpG5	cg00600454	0.75	0.67	0.64	0.04811	31.24	0.74	0.68
	Mean CpG1-CpG5		0.75	0.69	0.64	0.03933	43.48	0.75	0.69
**2**	CpG1	cg09310348	0.75	0.64	0.72	0.00042	25.39	0.74	0.66
	CpG2	N/A ^b^	0.81	0.67	0.73	0.00023	26.43	0.79	0.69
	Mean CpG1-CpG2		0.83	0.64	0.73	0.00025	25.67	0.68	0.81
**3**	CpG1	N/A ^b^	0.78	0.67	0.69	0.00502	28.15	0.76	0.68
	CpG2	N/A ^b^	0.69	0.72	0.68	0.00787	50.14	0.72	0.69
	CpG3	cg10121788	0.75	0.69	0.69	0.00557	34.28	0.75	0.69
	Mean CpG1-CpG3		0.78	0.67	0.68	0.00660	31.67	0.76	0.68
**4**	CpG1	cg14066163	0.81	0.59	0.69	0.00353	62.89	0.77	0.64
	CpG2	cg18455472	0.69	0.64	0.69	0.00359	51.92	0.69	0.64
	Mean CpG1-CpG2		0.83	0.56	0.69	0.00317	50.13	0.79	0.64
**5**	CpG1	cg19930657	0.75	0.72	0.76	0.00353	44.15	0.77	0.64

^a^ Human GRCh37/hg19 assembly; ^b^ CpG dinucleotide not annotated in GRCh37/hg19 database; ^c^ AUC—area under the curve; ^d^ Cutoff—methylation value above which a given sample is classified as fast-growing tumor; ^e^ NPV—negative predictive value; ^f^ PPV—positive predictive value.

**Table 3:** Two logistic regression models—comparison of full and final model.

	Sensitivity	Specificity	Cutoff	NPV	PPV	AUC (Bias)	Bootstrap AUC	AIC
**Full model**	0.81	0.82	0.53	0.82	0.8	0.858 (0.11)	0.744	99.006
**Final model**	0.78	0.90	0.57	0.81	0.87	0.845 (0.04)	0.806	87.421

## Data Availability

The datasets generated during the current study are not publicly available due to other ongoing studies but are available from the corresponding author on reasonable request.

## Supplementary Materials

The following supporting information can be downloaded at www.mdpi.com/article/10.3390/ijms23115962/s1.
