# Supplementary material for: Global DNA Methylation Profiling Reveals Differentially Methylated CpGs between Salivary Gland Pleomorphic Adenomas with Distinct Clinical Course
Source: Int J Mol Sci. 2022 May 25;23(11):5962. doi: 10.3390/ijms23115962 (PMC9180868; doi:10.3390/ijms23115962)
Supplement: Supplementary file 1 [file ijms-23-05962-s001.zip › Supplementary Figures.pdf]

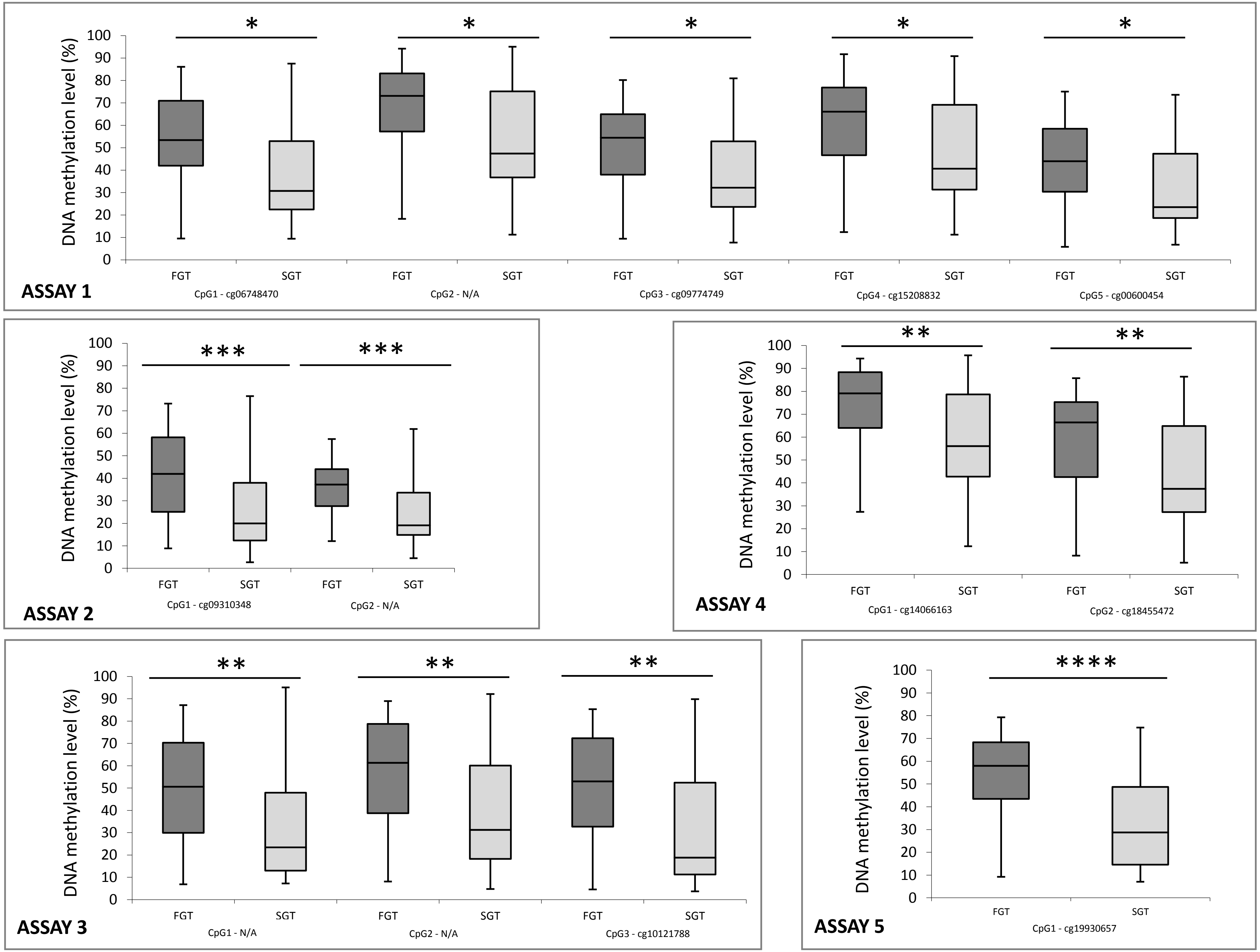

Supplementary Figure S1. Mean methylation differences between fast-growing (dark grey) and slow-growing (light grey) tumors for each CG in given assay by DNA bisulfite pyrosequencing.
The obtained p values were assigned with asterisks, as follows: * $p \leq 0.05$; ** $p \leq 0.01$; *** $p \leq 0.001$; **** $p \leq 0.0001$.
N/A - CpG dinucleotide not annotated in GRCh37/hg19 database

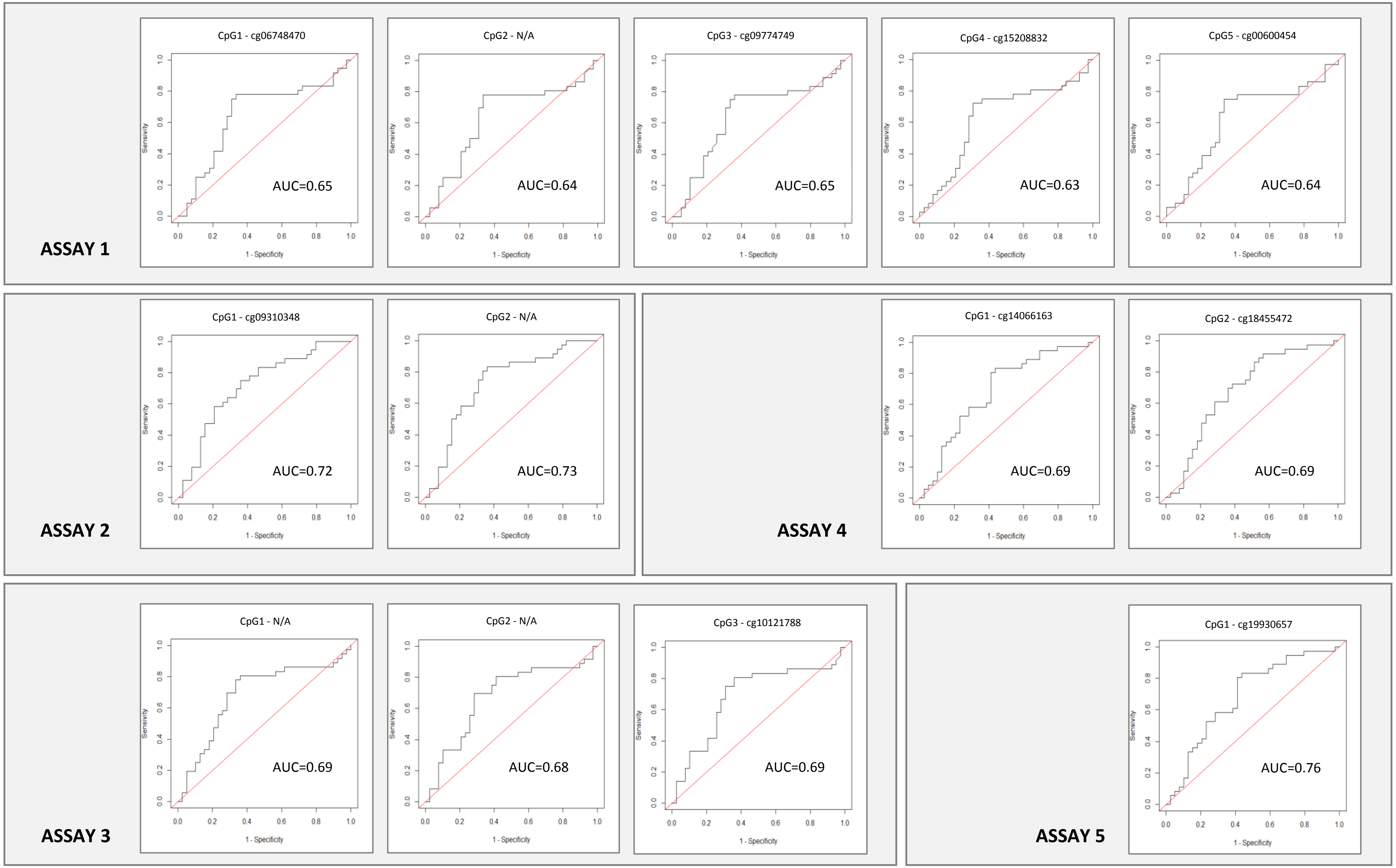

Supplementary Figure S2. ROC curves for individual CpGs in all 5 assays. AUC values are provided. N/A - CpG dinucleotide not annotated in GRCh37/hg19 database
