# Supplementary material for: Global DNA Methylation Profiling Reveals Differentially Methylated CpGs between Salivary Gland Pleomorphic Adenomas with Distinct Clinical Course
Source: Int J Mol Sci. 2022 May 25;23(11):5962. doi: 10.3390/ijms23115962 (PMC9180868; doi:10.3390/ijms23115962)
Supplement: Supplementary file 1 [file ijms-23-05962-s001.zip › Supplementary Table S1.pdf]

Table S1. The list of CpG tags differentiating between fast-growing tumors (FGT) and slow-growing tumors (SGT)

CG name	Chromosome (GRCh37/hg19)	Position (GRCh37/hg19)	MMV in FGT	SD (FGT)	MMV in SGT	SD (SGT)	MMD (FGT-SGT)
cg19312427	chr1	198174503	0.76	0.10	0.36	0.06	0.40
cg05822335	chr1	171179875	0.58	0.15	0.18	0.09	0.41
cg13548810	chr1	55349365	0.87	0.07	0.45	0.10	0.41
cg06748470	chr1	65467980	0.84	0.09	0.42	0.11	0.42
cg21159128	chr1	54693933	0.75	0.13	0.32	0.18	0.43
cg17361569	chr1	35965792	0.88	0.04	0.41	0.19	0.47
cg08308801	chr10	119867973	0.79	0.06	0.32	0.12	0.46
cg26508239	chr10	29759299	0.85	0.14	0.43	0.15	0.42
cg06838584	chr11	2504581	0.73	0.12	0.30	0.11	0.43
cg19345602	chr11	125615388	0.76	0.08	0.35	0.19	0.41
cg15175377	chr11	68920434	0.78	0.05	0.22	0.20	0.57
cg00143986	chr12	131606752	0.93	0.01	0.24	0.04	0.69
cg13930596	chr12	115108282	0.69	0.01	0.17	0.06	0.52
cg01078446	chr12	115113212	0.77	0.09	0.36	0.07	0.41
cg23764766	chr12	1459744	0.63	0.15	0.17	0.10	0.46
cg05950157	chr12	130603995	0.81	0.14	0.40	0.11	0.41
cg25428389	chr12	49277498	0.85	0.06	0.41	0.15	0.44
cg04660812	chr12	76054210	0.73	0.09	0.31	0.16	0.42
cg12496211	chr12	2193060	0.79	0.05	0.31	0.16	0.48
cg10731648	chr12	46745936	0.75	0.06	0.34	0.17	0.41
cg16406892	chr12	115118777	0.88	0.01	0.46	0.17	0.41
cg24009544	chr12	121613464	0.77	0.13	0.29	0.17	0.48
cg24401193	chr13	110996727	0.81	0.04	0.33	0.15	0.48
cg09310348	chr14	77371524	0.66	0.05	0.20	0.03	0.46
cg13261106	chr14	55999890	0.69	0.16	0.28	0.05	0.41
cg01333616	chr14	75722234	0.67	0.06	0.22	0.11	0.46
cg09964732	chr14	94998884	0.70	0.05	0.28	0.12	0.42
cg14496909	chr15	101729391	0.77	0.16	0.35	0.04	0.42
cg20661290	chr15	69545572	0.68	0.10	0.27	0.07	0.40
cg13271663	chr15	70387303	0.63	0.14	0.23	0.13	0.41
cg27554954	chr15	60691595	0.81	0.06	0.36	0.14	0.45
cg08884974	chr15	31505024	0.83	0.05	0.38	0.16	0.45
cg19335742	chr15	40566880	0.85	0.07	0.40	0.18	0.45
cg10121788	chr16	77469583	0.62	0.12	0.12	0.03	0.50
cg08418279	chr16	49733170	0.75	0.10	0.34	0.12	0.41
cg09417209	chr16	11715746	0.70	0.08	0.18	0.16	0.52
cg08249296	chr16	77469710	0.71	0.10	0.19	0.17	0.52
cg07749951	chr16	79352436	0.82	0.10	0.41	0.20	0.40
cg27419648	chr17	4757968	0.79	0.16	0.37	0.06	0.41
cg18455472	chr17	17311855	0.82	0.04	0.35	0.12	0.47
cg12968741	chr17	830967	0.83	0.02	0.36	0.17	0.47
cg14308452	chr19	5784184	0.82	0.08	0.42	0.19	0.40
cg15903609	chr2	148207352	0.83	0.05	0.35	0.07	0.48
cg06749642	chr2	114086683	0.75	0.08	0.33	0.08	0.42
cg08946713	chr2	191844998	0.66	0.19	0.18	0.08	0.48

cg04716277	chr2	219625165	0.83	0.05	0.43	0.16	0.40
cg01549082	chr2	20350783	0.66	0.16	0.24	0.17	0.42
cg26035105	chr2	121105464	0.71	0.04	0.30	0.20	0.42
cg13170954	chr20	40619155	0.79	0.08	0.36	0.20	0.43
cg23996696	chr21	31322913	0.88	0.03	0.26	0.04	0.62
cg01416479	chr21	47796873	0.91	0.03	0.44	0.07	0.47
cg06684407	chr21	46356806	0.83	0.12	0.41	0.08	0.42
cg01111856	chr22	46508451	0.92	0.02	0.46	0.16	0.46
cg24971189	chr3	71294237	0.75	0.16	0.28	0.03	0.48
cg03072598	chr3	71294380	0.77	0.08	0.23	0.05	0.53
cg22712970	chr3	71106959	0.76	0.20	0.31	0.10	0.45
cg15635553	chr3	195339050	0.88	0.03	0.34	0.11	0.53
cg19930657	chr3	171529610	0.82	0.01	0.31	0.16	0.51
cg02505331	chr4	96560436	0.76	0.06	0.32	0.06	0.43
cg10925937	chr4	105890788	0.67	0.10	0.24	0.11	0.43
cg03804409	chr4	47216088	0.82	0.06	0.33	0.17	0.48
cg05983326	chr5	140710688	0.60	0.10	0.14	0.03	0.46
cg18361975	chr5	106738731	0.76	0.12	0.34	0.05	0.41
cg08400424	chr5	142357042	0.74	0.04	0.33	0.14	0.41
cg24408662	chr5	103079926	0.77	0.09	0.36	0.14	0.41
cg24874236	chr5	25490530	0.66	0.13	0.26	0.19	0.40
cg25001484	chr6	20302622	0.82	0.01	0.26	0.00	0.56
cg04474876	chr6	126193612	0.63	0.20	0.21	0.02	0.41
cg21754524	chr6	149175336	0.68	0.12	0.24	0.08	0.43
cg12801037	chr6	11793280	0.82	0.12	0.40	0.09	0.42
cg08570639	chr6	113371757	0.68	0.06	0.25	0.10	0.43
cg12422716	chr6	111968507	0.81	0.07	0.40	0.15	0.41
cg10969178	chr6	31838402	0.82	0.16	0.40	0.18	0.42
cg00869930	chr6	156904035	0.84	0.08	0.43	0.18	0.41
cg14815476	chr6	158463428	0.89	0.03	0.48	0.19	0.41
cg06583111	chr7	28575984	0.76	0.09	0.32	0.09	0.44
cg20819752	chr7	112060391	0.77	0.19	0.35	0.13	0.42
cg08961454	chr8	117243882	0.76	0.08	0.33	0.05	0.43
cg13788620	chr8	51306508	0.57	0.09	0.16	0.07	0.41
cg02323453	chr8	101475221	0.68	0.07	0.27	0.08	0.41
cg04651963	chr8	36896788	0.71	0.14	0.30	0.14	0.40
cg27606001	chr8	17549898	0.79	0.14	0.36	0.17	0.43
cg01127608	chr9	129377854	0.53	0.10	0.06	0.01	0.47
cg04996334	chr9	129381005	0.79	0.16	0.21	0.14	0.58
cg14635269	chr9	129439339	0.77	0.01	0.35	0.18	0.42

Analysis performed with the use of Infinium Human Methylation EPIC 850k BeadChip Array (Illumina). MMV - mean methylation value; SD - standard deviation; MMD - mean methylation difference
