# Supplementary material for: Global DNA Methylation Profiling Reveals Differentially Methylated CpGs between Salivary Gland Pleomorphic Adenomas with Distinct Clinical Course
Source: Int J Mol Sci. 2022 May 25;23(11):5962. doi: 10.3390/ijms23115962 (PMC9180868; doi:10.3390/ijms23115962)
Supplement: Supplementary file 1 [file ijms-23-05962-s001.zip › Supplementary Table S2.pdf]

Table S2. The characteristics of 5 regions selected for DNA bisulfite pyrosequencing

Region	CG name (GRCh37/hg19)	Chromosome (GRCh37/hg19)	Position (GRCh37/hg19)	Region length (bp)	Fast-growing tumors						Slow-growing tumors						MMD (FGT-SGT)
					FGT-1	FGT-2	FGT-3	FGT-4	MMV in FGT	SD (FGT)	SGT-1	SGT-2	SGT-3	SGT-4	MMV in SGT	SD (SGT)	
**1**	**cg06748470**	chr1	**65467980**	38	**0.74**	**0.79**	**0.95**	**0.88**	**0.84**	**0.09**	**0.52**	**0.27**	**0.41**	**0.47**	**0.42**	**0.11**	**0.42**
	cg09774749		65467991		0.61	0.6	0.79	0.71	0.68	0.09	0.45	0.32	0.4	0.42	0.4	0.06	0.28
	cg15208832		65467997		0.64	0.6	0.83	0.76	0.71	0.1	0.46	0.32	0.38	0.41	0.39	0.06	0.32
	cg00600454		65468017		0.46	0.46	0.63	0.61	0.54	0.09	0.31	0.21	0.31	0.29	0.28	0.05	0.26
**2**	cg18413218	chr14	77371470	55	0.41	0.56	0.6	0.61	0.54	0.09	0.2	0.16	0.21	0.21	0.19	0.02	0.35
	**cg09310348**		**77371524**		**0.62**	**0.61**	**0.71**	**0.67**	**0.66**	**0.05**	**0.18**	**0.16**	**0.23**	**0.21**	**0.2**	**0.03**	**0.46**
**3**	**cg10121788**	chr16	**77469583**	128	**0.5**	**0.57**	**0.77**	**0.65**	**0.62**	**0.12**	**0.15**	**0.09**	**0.13**	**0.11**	**0.12**	**0.03**	**0.5**
	**cg08249296**		**77469710**		**0.57**	**0.71**	**0.8**	**0.77**	**0.71**	**0.1**	**0.03**	**0.13**	**0.19**	**0.42**	**0.19**	**0.17**	**0.52**
**4**	**cg18455472**	chr17	**17311855**	12	**0.78**	**0.86**	**0.8**	**0.84**	**0.82**	**0.04**	**0.42**	**0.45**	**0.35**	**0.17**	**0.35**	**0.12**	**0.47**
	cg14066163		17311866		0.85	0.85	0.85	0.87	0.86	0.01	0.57	0.7	0.54	0.25	0.52	0.19	0.34
**5**	cg14000730	chr3	171529077	534	0.49	0.43	0.55	0.65	0.53	0.09	0.28	0.22	0.2	0.28	0.24	0.04	0.29
	cg26752773		171529090		0.79	0.69	0.71	0.76	0.74	0.05	0.61	0.22	0.23	0.32	0.34	0.19	0.39
	**cg19930657**		**171529610**		**0.83**	**0.81**	**0.81**	**0.83**	**0.82**	**0.01**	**0.49**	**0.23**	**0.13**	**0.38**	**0.31**	**0.16**	**0.51**

Primarily selected six CpGs are bolded. The remaining CpGs are located in close proximity. MMV - mean methylation value; SD - standard deviation; MMD - mean methylation difference
