# Supplementary material for: Global DNA Methylation Profiling Reveals Differentially Methylated CpGs between Salivary Gland Pleomorphic Adenomas with Distinct Clinical Course
Source: Int J Mol Sci. 2022 May 25;23(11):5962. doi: 10.3390/ijms23115962 (PMC9180868; doi:10.3390/ijms23115962)
Supplement: Supplementary file 1 [file ijms-23-05962-s001.zip › Supplementary Table S3.pdf]

Table S3. The genomic coordinates of CpG dinucleotides included in each pyrosequencing assay

**Pyrosequencing assay no.**	**CpG no.**	**CpG name**	**Chromosomal localization (GRCh37/hg19)**
**1**	CpG1	cg06748470	chr1:65,467,980-65,467,981
	CpG2	N/A	chr1:65,467,986-65,467,987
	CpG3	cg09774749	chr1:65,467,991-65,467,992
	CpG4	cg15208832	chr1:65,467,997-65,467,998
	CpG5	cg00600454	chr1:65,468,017-65,468,018
**2**	CpG1	cg09310348	chr14:77,371,524-77,371,525
	CpG2	N/A	chr14:77,371,501-77,371,502
**3**	CpG1	N/A	chr16:77,469,605-77,469,606
	CpG2	N/A	chr16:77,469,589-77,469,590
	CpG3	cg10121788	chr16:77,469,583-77,469,584
**4**	CpG1	cg14066163	chr17:17,311,866-17,311,867
	CpG2	cg18455472	chr17:17,311,855-17,311,856
**5**	CpG1	cg19930657	chr3:171,529,610-171,529,611

N/A - CpG dinucleotide not annotated in GRCh37/hg19 database.
In assay number 5, only one tag (cg19930657) was analyzed, due to the long distance from other adjacent tags with similar methylation pattern.
