# Supplementary material for: Global DNA Methylation Profiling Reveals Differentially Methylated CpGs between Salivary Gland Pleomorphic Adenomas with Distinct Clinical Course
Source: Int J Mol Sci. 2022 May 25;23(11):5962. doi: 10.3390/ijms23115962 (PMC9180868; doi:10.3390/ijms23115962)
Supplement: Supplementary file 1 [file ijms-23-05962-s001.zip › Supplementary Table S4.pdf]

Table S4. The clinical characteristics of PA patients

	Total	FGT	SGT
Number			
	75	36	39
Age (years)			
Median	43	43	44
Range	15-76	15-76	22-67
Sex			
Female	48	21	27
Male	27	15	12
Treatment			
ECD	9	5	4
Partial superficial parotidectomy	12	6	6
Superficial parotidectomy	21	9	12
Subtotal parotidectomy	16	10	6
Total parotidectomy	8	4	4
SGD	9	2	7

ECD - extracapsular disection

SGD - salivary gland dissection
