# Supplementary material for: Global DNA Methylation Profiling Reveals Differentially Methylated CpGs between Salivary Gland Pleomorphic Adenomas with Distinct Clinical Course
Source: Int J Mol Sci. 2022 May 25;23(11):5962. doi: 10.3390/ijms23115962 (PMC9180868; doi:10.3390/ijms23115962)
Supplement: Supplementary file 1 [file ijms-23-05962-s001.zip › Supplementary Table S5.pdf]

Table S5. The characteristics of the used pyrosequening assays

Pyrosequencing assay no.	Primer	Primer sequence 5'-3'	Annealing temperature	Product size (bp)	Localization (GRCh37/hg19)	Number of analyzed CpG
**1**	F	GGGTAAATGAGTGAGTTTTAGTTAGAA	53°C	141	chr1:65,467,920-65,468,060	5
	R	biotin-CCCTTAAATCAACCACTAAATAACTT				
	S	TTGTTTTTGGGTAGTGGG	Sequencig primer			
**2**	F	biotin-GAAGTAATGTTTTAAGGGTATTGG	55°C	173	chr14:77,371,429-77,371,601	2
	R	CCAACCTACTAATTACTAAACATTTTATAC				
	S	ACTCTACACTTAATAACTA	Sequencig primer			
**3**	F	TTGGGTATTTGGGGTTAAGATTTAA	55°C	146	chr16:77,469,489-77,469,634	3
	R	biotin-ACCAAAACAATAAACTATCACTATCTAC				
	S	GGTATTTGGGGTTAAGATTTAAT	Sequencig primer			
**4**	F	AGGATTGTTTATAGATATTAAGGTTATGAA	55°C	94	chr17:17,311,818-17,311,911	2
	R	biotin-ATTCTTTCTCATTTCCAAATCTCAC				
	S	ATAGATATTAAGGTTATGAAAGATA	Sequencig primer			
**5**	F	GTTATAGATGGTTTGATAAGTTTTGTGT	56°C	153	chr3:171,529,577-171,529,729	1
	R	biotin-ACAAATACCAAATATTTTCATAATAACAC				
	S	GGTTTGATAAGTTTTGTGTA	Sequencig primer			

F-forward primer; R-reverse primer; S-sequencing primer
