# Supplementary material for: Global DNA Methylation Profiling Reveals Differentially Methylated CpGs between Salivary Gland Pleomorphic Adenomas with Distinct Clinical Course
Source: Int J Mol Sci. 2022 May 25;23(11):5962. doi: 10.3390/ijms23115962 (PMC9180868; doi:10.3390/ijms23115962)
Supplement: Supplementary file 1 [file ijms-23-05962-s001.zip › Supplementary Table S6.pdf]

Table S6. The composition of PCR reaction mixture (A) and the PCR reaction conditions (B)

A

**PCR reaction mixture**	**Total vol - 25 ul**
	1x PyroMark Master Mix (HotStarTaq DNA Polymerase, dNTPs, 1x PyroMark PCR Buffer)
	10 pmol of each primer
	1x Coral Load Concentrate
	1 ul of converted DNA

B

	**Step**	**Temp [°C]**	**Time**	
**Pyrosequencing PCR conditions**	Initial heat activation	95	15 min.	
	Denaturation	94	30 sec.	45 cycles
	Annealing	53-56*	30 sec.	
	Extension	72	30 sec.	
	Final extension	72	10 min.	

*The annealing temperature depends on the used primers
